# Cardiovascular magnetic resonance-guided right heart catheterization in a conventional CMR environment – predictors of procedure success and duration in pulmonary artery hypertension

**DOI:** 10.1186/s12968-019-0569-9

**Published:** 2019-09-09

**Authors:** Daniel S. Knight, Tushar Kotecha, Ana Martinez-Naharro, James T. Brown, Michele Bertelli, Marianna Fontana, Vivek Muthurangu, J. Gerry Coghlan

**Affiliations:** 1 National Pulmonary Hypertension Service, Royal Free London NHS Foundation Trust, Pond Street, London, NW3 2QG UK; 2 Department of Cardiology, Royal Free London NHS Foundation Trust, Pond Street, London, NW3 2QG UK; 3 UCL Department of Cardiac MRI, University College London (Royal Free Campus), Rowland Hill Street, London, NW3 2PF UK; 4 Centre for Cardiovascular Imaging, Institute of Cardiovascular Science, University College London and Great Ormond Street Hospital for Children, 30 Guilford Street, London, WC1N 1EH UK

**Keywords:** Cardiovascular magnetic resonance imaging, Guidewire, Interventional cardiovascular MR catheterization, Pulmonary hypertension, Right heart catheterization

## Abstract

**Background:**

Cardiovascular magnetic resonance imaging (CMR) is valuable for the investigation and management of pulmonary artery hypertension (PAH), but the direct measurement of pulmonary hemodynamics by right heart catheterization is still necessary. CMR-guided right heart catheterization (CMR-RHC) combines the benefits of CMR and invasive cardiac catheterization, but its feasibility in patients with acquired PAH has not been established. The aims of this study are to: (1) demonstrate the feasibility of CMR-RHC in patients being assessed for PAH in a conventional diagnostic CMR scanner room; (2) determine the predictors of (i) procedure duration, and (ii) procedural failure or technical difficulty as determined by the adjunctive need for a guidewire.

**Methods:**

Fifty patients investigated for suspected or known PH underwent CMR-RHC. Durations of separate procedural components were recorded, including time taken to pass the catheter from the femoral vein to a stable wedge position (procedure time) and total time the patient spent in the CMR department (department time). Associations between procedural failure/guidewire usage and hemodynamic/CMR measures were assessed using logistic regression. Relationships between procedure times and hemodynamic/CMR measures were evaluated using Spearman’s correlation coefficient.

**Results:**

A full CMR-RHC study was successfully completed in 47 (94%) patients. CMR-conditional guidewires were used in 6 (12%) patients. Metrics associated with guidewire use/procedural failure were higher mean pulmonary artery (PA) pressures (mPAP: OR = 1.125, *p* = 0.018), right heart dilatation (right ventricular (RV) end-systolic volume (RVESV): OR = 1.028, *p* = 0.018), RV hypertrophy (OR = 1.050, *p* = 0.0067) and RV ejection fraction (EF) (OR = 0.914, *p* = 0.014). Median catheter and department times were 3.6 (2.0–7.7) minutes and 60.0 (54.0–68.5) minutes, respectively. All procedure times became significantly shorter with increasing procedural experience (*p* < 0.05). Catheterization time was also associated with PH severity (RV systolic pressure: rho = 0.46, *p* = 0.0013) and increasing RV end-systolic volume (RVESV: rho = 0.41, *p* = 0.0043), hypertrophy (rho = 0.43, *p* = 0.0025) and dysfunction (RVEF: rho = − 0.32, *p* = 0.031).

**Conclusions:**

This study demonstrates that CMR-RHC using standard technology can be incorporated into routine clinical practice for the investigation of PAH. Procedural failure was rare but more likely in patients with severe PAH. Procedure time is clinically acceptable and increases with worsening PAH severity.

**Electronic supplementary material:**

The online version of this article (10.1186/s12968-019-0569-9) contains supplementary material, which is available to authorized users.

## Background

Cardiovascular magnetic resonance (CMR) is a valuable imaging technique for the investigation and management of patients with pulmonary artery (PA) hypertension (PAH). It provides highly accurate measurements of right ventricular (RV) size, systolic function and mass, all of which are prognostic [1, 2]. However, invasive catheter-derived measurement of pulmonary artery pressure and calculation of pulmonary vascular resistance (PVR) are still necessary for optimum management of PAH.

One way of combining the benefits of CMR with catheter-derived pressure measurement is CMR-guided right heart catheterization (CMR-RHC). This was first demonstrated in humans over 15 years ago by Razavi et al., and has been replicated in several centres worldwide [3–7]. This technique has several advantages over conventional cardiac catheterization including: improved soft tissue visualization, reduced exposure to ionizing radiation and reference standard evaluation of physiology. Nevertheless, CMR-RHC still has not entered mainstream clinical practice. One possible reason was that early studies were performed in hybrid CMR/X-ray fluoroscopic suites and often used both imaging modalities [6]. This is a barrier to adoption due to the high cost and space requirements of such suites. However, more recent studies have shown that right heart catheterization is possible using CMR alone in most patients [5, 7]. Thus, it is now possible to incorporate CMR-RHC into routine clinical practice in a conventional CMR suite.

Therefore, we set up a clinical CMR-RHC service at one of the United Kingdom national PAH centres. The aims of this study are: (1) to demonstrate the feasibility of CMR-RHC in an unselected population of patients being assessed for suspected or known PAH in a conventional diagnostic CMR scanner room, and (2) to determine the predictors of (i) procedure duration, and (ii) procedural failure or technical difficulty as determined by the adjunctive need for a guidewire.

## Methods

### Patient population

CMR-guided RHC was approved by the Royal Free London NHS Foundation Trust New Interventional Procedures Committee in November 2017. Consecutive patients referred for RHC at the National PH Service at the Royal Free London NHS Foundation Trust between November 2017 and October 2018 and who were available to attend within the timeframe of newly designated CMR-RHC sessions at our institution were offered to undergo CMR-RHC. The clinical indication for CMR-RHC was the same as that for conventional X-ray fluoroscopy-guided RHC, specifically the investigation of suspected or known PH. In particular, patients with scleroderma were referred for CMR-RHC either for the investigation of symptomatic dyspnea and/or using the DETECT algorithm, a systematic clinical screening program used for the evidence-based detection of PAH in this condition [8]. Exclusion criteria for CMR-RHC were arrhythmia, patients requiring an additional X-ray fluoroscopy-guided procedure (including coronary angiography or conventional pulmonary angiography), pregnancy and contraindications to CMR. All ethics were approved by the UCL/UCLH Joint Committees on the Ethics of Human Research Committee, and all participants provided written informed consent.

### CMR-RHC suite set-up

**Fig. 1:**
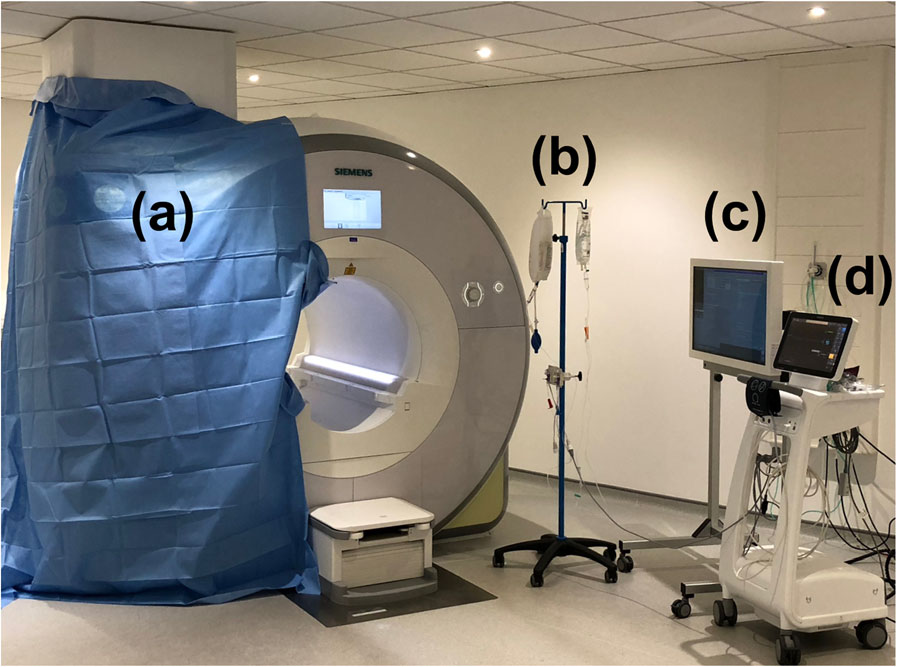
Cardiovascular magnetic resonance-right heart catheterization (CMR-RHC) procedure room equipment set-up. **a** 1.5 T CMR scanner with one large sterile drape attached to the front and side of the scanner, and one small sterile drape attached to the front of the scanner and passing into the bore. **b** CMR compatible drip stand with pressure transducer. **c** CMR-compatible monitor to mirror the scanner display for in-room catheter visualization. **d** CMR-compatible blood pressure monitor (displaying invasive and non-invasive blood pressure measurements along with pulse oximetry)

All procedures were performed in a suite equipped with a 1.5 T CMR scanner (Magnetom Aera, Siemens Healthcare, Erlangen, Germany). A 40-in. CMR-compatible monitor (NordicNeuroLab AS, Bergen, Norway) was used to mirror the scanner display for in-room catheter visualization. A CMR-compatible blood pressure monitor (Expression MR400 or Expression IP5, Invivo International, Philips Healthcare, Best, The Netherlands) was used to measure invasive pressures with the transducers attached to a CMR-compatible drip stand (Fig. 1). Patients receiving continuous parenteral prostanoid therapy had their infusion administered using a volumetric infusion pump (Infusomat Space pump, B. Braun Medical Ltd., Sheffield, United Kingdom) placed inside a unit with shielded aluminium housing (SpaceStation MRI, B. Braun Medical Ltd., Sheffield, United Kingdom).

The staff present for the procedure included an interventional cardiologist, imaging cardiologist, CMR radiographer, cardiac physiologist and a second operator in the scanner room for patient nursing and to assist the interventional operator when required.

### Patient preparation

Patients initially lay on the scanner table to have surface vector-cardiogram (VCG) electrodes attached and a phased array body coil positioned. The scanner table was then undocked and transferred to the preparation area just outside the scanner room door (Additional file 1: Video S1).

Patients undergoing CMR-RHC were offered the option of mild sedation using intravenous benzodiazepines prior to venous sheath insertion. Vascular access was obtained under local anaesthetic using either manual palpation or under ultrasound guidance (Additional file 1: Video S2). The right femoral vein was chosen as the preferred route of access due to our conventional cardiac catheterization laboratory standard operating procedures, with right internal jugular access used in cases in which femoral venous access was not feasible. In order to maintain sterility of the operating field, the sterile drape was folded over onto the patient firstly from the operator side and subsequently from the opposite side of the table (Additional file 1: Video S3). At this time, the side of the scanner and bore closest to the operator were also draped (Fig. 1). The patient was then transferred back into the CMR scanner room and the table was docked (Additional file 1: Video S4). A non-invasive blood pressure cuff (set to automatically measure at 5-min intervals) and a peripheral oxygen saturation finger probe were attached to the patient. The patient is then positioned inside the scanner bore, following which the drapes are unfolded whilst maintaining sterility.

The catheter operator used the patient’s headphones, allowing communication with the scanner operator. The patient used standard CMR ear defenders and had an alarm to activate if they wished to speak to the team during the procedure in addition to regularly being verbally checked upon by a staff member in the scanner room.

### CMR-guided right heart catheterization

#### Preliminary imaging

**Fig. 2:**
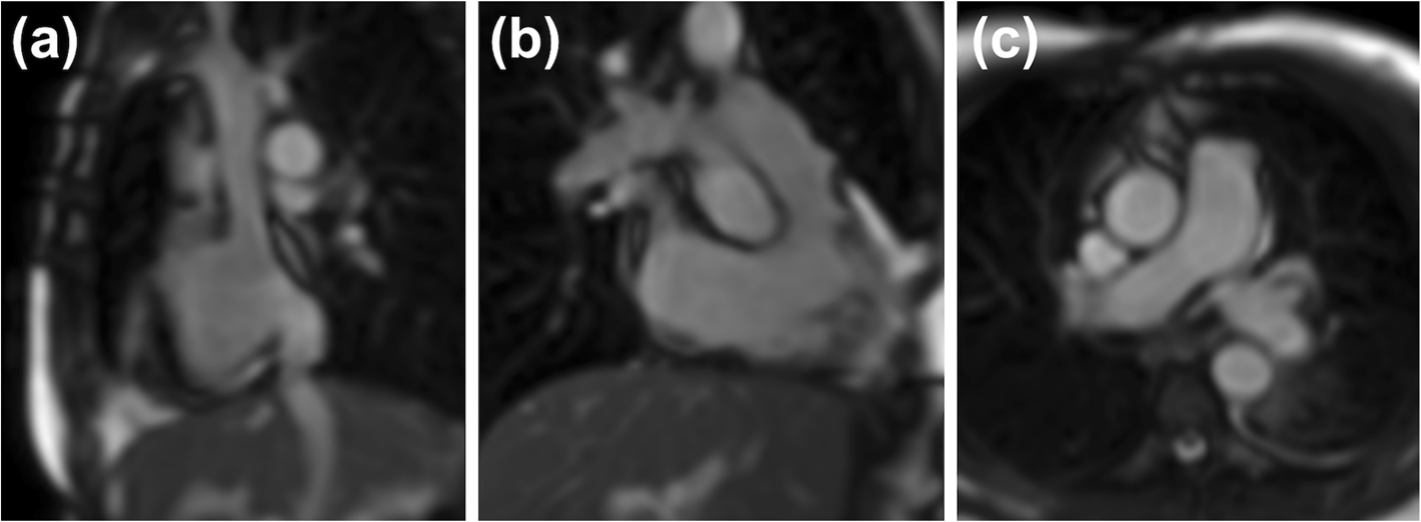
Three reference views acquired using the interactive real-time sequence. **a** Bicaval view, **b** Right ventricular long axis (RVLA) view, **c** Main pulmonary artery (PA) bifurcation view

After conventional localizers, an interactive real-time sequence (that was subsequently also used for catheter visualization) was used to plan three specific reference views. These were the bicaval view, the RV long axis (RVLA) view and the main PA bifurcation view (Fig. 2).

#### Catheter visualization and guidance

**Fig. 3:**
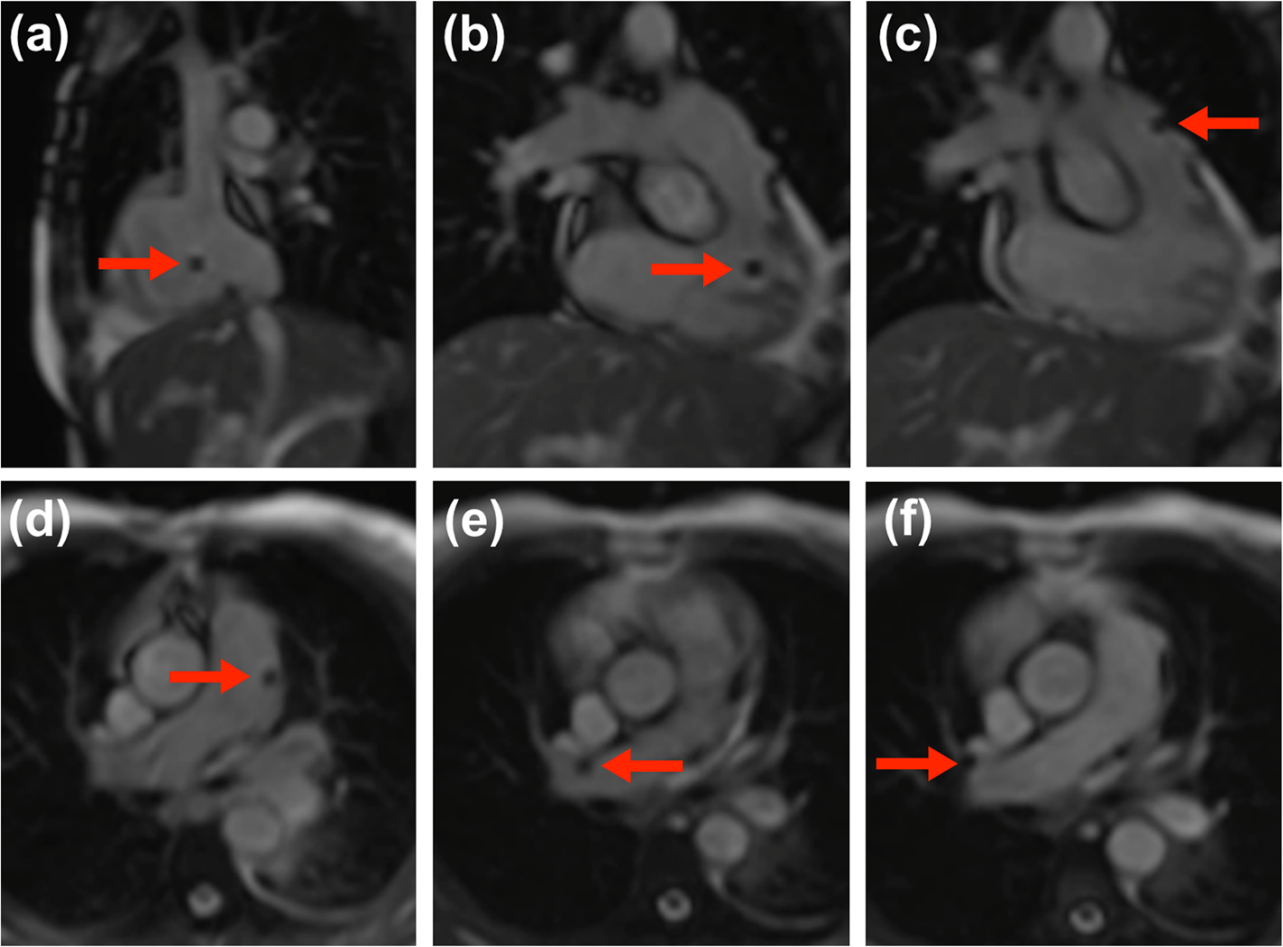
The air-filled balloon of the balloon wedge-pressure end-hole catheter visualized as a signal void passing through the right heart, pulmonary artery and finally to the wedge position under CMR visualization. Here, the balloon is seen in the **a** right atrium, **b** right ventricle, **c** right ventricular outflow tract, **d** main pulmonary artery, **e** right branch pulmonary artery approaching a wedge position, **f** wedge position


A 6 French 110-cm single lumen balloon wedge-pressure end-hole catheter (Arrow, Teleflex, Buckinghamshire, United Kingdom) was used for all catheterizations. The balloon was filled with air during catheterization, allowing the catheter tip to be visualized as a signal void (Fig. 3). The catheter was advanced from the groin through the right heart, pulmonary artery and finally to the wedge position under CMR visualization. The interactive real-time sequence that was used allowed the catheter to be ‘tracked’ by switching between the previously stored reference views with real-time adjustments performed by the imaging cardiologist (Additional file 2: Video S5). The interactive real-time sequence parameters typically used were those as supplied by the manufacturer: TR/TE 2.4 ms/0.98 ms; flip angle 50^o^; bandwidth 1002 Hz/pixel; FOV 320 × 320 mm; matrix 128 × 128 pixels; slice thickness 10 mm; GRAPPA rate 2; voxel size 2.5 × 2.5 mm, temporal resolution 123 ms, 8 frames/s). The only sequence parameter modified by the scan operator was the field of view when required according to patient habitus.

**Additional file 2: Video S5** Interactive real-time CMR-guidance of the air-filled balloon of the balloon wedge-pressure end-hole catheter. This is visualized as a signal void passing through the right heart, pulmonary artery and finally to the wedge position. The interactive real-time sequence allows the catheter to be ‘tracked’ by switching between the previously stored reference views with real-time adjustments. (MOV 11903 kb)


Two different types of CE-marked 0.035″ diameter CMR conditional guidewires were available during the study period, namely the Emery Glide Straight tip MR Wire (Nano4Imaging GmbH, Aachen, Germany) and the MRline guidewire (EPflex Feinwertechnik GmbH, Dettingen an der Erms, Germany). The choice of guidewire was determined by product availability (in five cases) and operator discretion (in one case). The proximal portion of both guidewires were equipped with CMR visible markers allowing visualization as additional image artefacts around the passive markers.

#### CMR hemodynamic assessment

Once in the wedge position, both pulmonary capillary wedge pressure (PCWP) and mean pulmonary artery pressure (mPAP) were measured. Flow data in the proximal ascending aorta, sub-pulmonary artery and right and left branch PA were acquired using a conventional free breathing velocity-encoded phase contrast sequence to derive cardiac output. This allowed calculation of PVR using the formula: PVR = (mPAP – PCWP) / cardiac output. A cine stack of short-axis slices for assessment of biventricular volumes along with a 4-chamber cine were then acquired. Cine imaging was performed using a breath-hold balanced steady-state free precession (bSSFP) sequence unless the patient had difficulty with breath-holding, at which point real-time cine imaging was utilized. All CMR imaging was performed using commercially available sequences. Tricuspid and pulmonary regurgitant fractions were calculated as described previously [1].

**Fig. 4:**
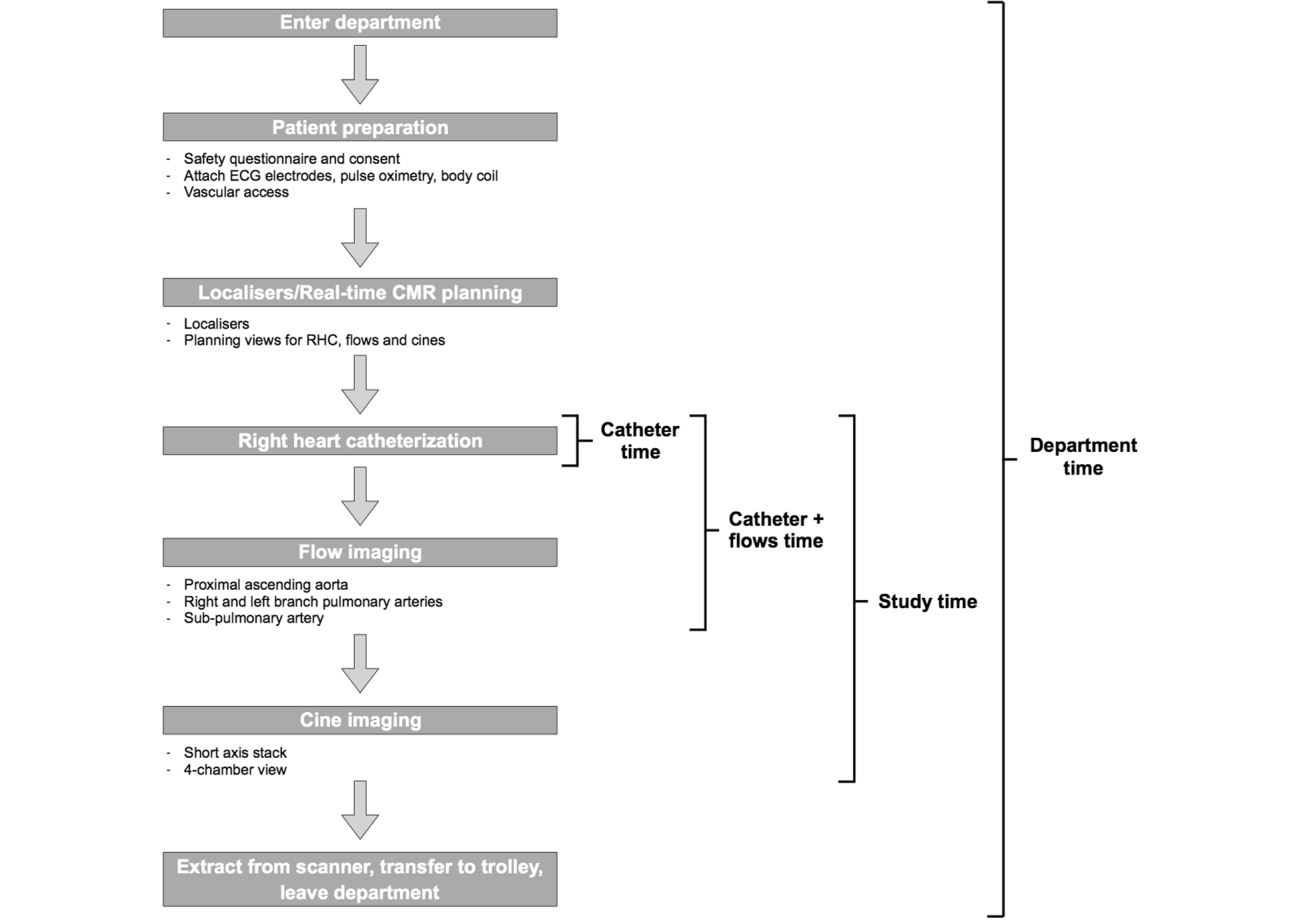
Workflow for a CMR-RHC study with four specific timed components of the study. Catheter time, catheter plus flows time, full CMR-RHC study time and the department time were all recorded

The full workflow for a CMR-RHC study is shown in Fig. 4, with four specific timed components of the study defined as follows:

- Catheter time represented the time taken from catheter insertion at the venous sheath to obtaining a stable pulmonary capillary wedge position. This includes catheter manipulation under real-time CMR-guidance and pressure recordings. Sampling of PA blood for measurement of oxygen saturations was performed as it has been shown to be prognostic independent of cardiac index [9] and is part of our standard PAH data set. In addition, oximetry was performed to confirm satisfactory wedge position in cases where the PCWP was ≥13 mmHg.
- Catheter and flows time included the catheter time plus the acquisition of phase contrast CMR data for proximal ascending aorta, sub-pulmonary artery and right and left branch PA flows.
- Study time was defined as the total time required for acquiring a complete CMR-RHC study dataset, including the time taken to acquire all cine imaging in addition to the catheter and flows time.
- Department time comprised the total time the patient spent in the CMR department, including all procedural preparation, acquisition of scout and localizer images, study time, extraction from the scanner and transfer out of the department to the recovery ward.

## Statistics

All statistical analyses were performed in R (Rstudio 1.14). Data were examined for normality using the Shapiro Wilk test. Normally distributed variables were expressed as mean ± standard deviation. Non-normally distributed variables were expressed as median (interquartile range). Proportions were expressed as percentages.

Procedural failure and guidewire usage were combined as an outcome variable due to significant overlap. Associations between this outcome and hemodynamic and CMR measures were assessed using logistic regression.

Spearman’s correlation coefficient was used to analyse the relationships between catheter time and hemodynamic and CMR measures, as well as all timing parameters and procedure number. A *p*-value < 0.05 was considered statistically significant.

## Results

### Patient demographics

Patient demographics are summarized in Table 1. The majority of the patient cohort (32, 64%) were in World Health Organisation (WHO) functional class III. The significant female preponderance of patients (80%) is reflective of the underlying disease processes in our cohort; 30 out of 40 female patients (75%) had connective tissue disease and seven (18%) had idiopathic PH.

**Table 1:** Patient demographics

Characteristic	Finding	Range (min-max)
Age (years)	57 +/− 15	20–83
Female gender, n (%)	40 (80)	
Height (m)	1.63 (1.56–1.73)	1.46–1.95
Weight (kg)	71 +/− 16	40–105
BSA (m^2^)	1.8 +/− 0.2	1.4–2.4
6MWD (metres)	333 (150–440)	0–582
NT-proBNP (ng/L)	320 (110–841)	50–52,746
WHO FC, n (%)		
1	2 (4)	
2	12 (24)	
3	32 (64)	
4	4 (8)	
PAH, n (%)	39 (78)	
Group 1	33 (66)	
- Idiopathic	8	
- CTD	23	
- PVOD	1	
- Portopulmonary	1	
Group 2	2 (4)	
Group 3	2 (4)	
Group 4	1 (2)	
Group 5	1 (2)	
PAH therapy, n (%)		
- Phosphodiesterase type 5 inhibitor	24 (48)	
- Endothelin receptor antagonist	23 (46)	
- Intravenous Epoprostenol	2 (4)	

All normally distributed data are presented as mean ± standard deviation, non-parametric data are presented as median (Q1-Q3) and proportions as n (%)

*BSA* Body surface area, *CTD* Connective tissue disease, *NT-proBNP* N-terminal pro-brain natriuretic peptide, *PAH* Pulmonary artery hypertension, *PVOD* Pulmonary veno-occlusive disease, *WHO FC* World Health Organisation Functional Class, *6MWD* 6-min walk distance

Twenty-six (52%) patients already had a known diagnosis of PH, the majority of whom (25, 96%) were already on pulmonary vasodilator therapy. This included 2 patients who were receiving continuous intravenous epoprostenol. Twenty-four patients had suspected PH and the CMR-RHC was their index invasive hemodynamic assessment. In this group, 13 patients had the diagnosis confirmed and 11 patients (10 with scleroderma and 1 with antisynthetase syndrome) had normal PA pressures. Invasive and CMR derived measures are shown in Table 2, demonstrating a wide spectrum of pulmonary hemodynamics and right heart sizes amongst the patient population.

**Table 2:** Right heart catheterization and CMR data or range

Variable	Finding	Range (min-max)
RVSP (mmHg)	59 (40–78)	24–132
RVDP (mmHg)	8 (6–12)	1–25
mPAP (mmHg)	35 +/− 14	12–80
PCWP (mmHg)	9 (8–11)	4–35
TPG (mmHg)	25 (13–35)	6–72
CO (L/min)	5.1 +/− 1.4	3.0–9.4
PVR (dynes.sec.cm^− 5^)	400 (197–615)	80–1340
RVEDV (mL)	135 +/− 37	51–243
RVESV (mL)	58 (43–75)	14–159
RVSV (mL)	70 +/− 19	36–121
RVEF (%)	54 +/− 13	20–76
RV mass (g)	69 +/− 28	20–142
RA area (cm^2^)	23 +/− 6	11–42
LVEDV (mL)	103 (82–131)	54–278
LVESV (mL)	36 (28–50)	15–203
LVSV (mL)	71 +/− 21	35–116
LVEF (%)	65 (60–71)	27–83
LV mass (g)	108 (88–135)	64–258
LA area (cm^2^)	22 +/− 6	10–36

All normally distributed data are presented as mean ± standard deviation, non-parametric data are presented as median (Q1-Q3)

*CO* Cardiac output, *EDV* End diastolic volume, *EF* Ejection fraction, *ESV* End systolic volume, *LA* Left atrium, *LV* Left ventricle, *LVEDV *left ventricular end-diastolic volume, *LVEF* left ventricular ejection fraction, *LVESV*  left ventricular end-systolic volume, *LVSV* left ventricular stroke volume, *mPAP* Mean pulmonary artery pressure, *PCWP* Pulmonary capillary wedge pressure, *PVR* Pulmonary vascular resistance, *RA* Right atrium, *RV* Right ventricle, *RVDP* Right ventricular diastolic pressure, *RVEDV* right ventricular end-diastolic volume, *RVESV* right ventricular end-systolic volume, *RVSP* Right ventricular systolic pressure, *SV* Stroke volume, *TPG* Transpulmonary gradient

### Procedural details and success

Forty-nine CMR-RHC studies were performed using femoral venous access and one study (patient number 16) was performed from the right internal jugular vein due to the presence of an inferior vena cava filter. The femoral vein was successfully identified and cannulated by manual palpation in 48 patients and using ultrasound guidance in 1 case; ultrasound guidance was used as the default technique for internal jugular venous access. There were no significant vascular complications, with a simple hematoma reported in one patient that was conservatively managed. Sedation was offered to all patients and subsequently used in 17 (34%) patients without complication; the remaining 33 (66%) of patients declined any sedation as per their preference. No patients requested the study to be terminated early.

A full CMR-RHC study was successfully completed in 47 (94%) patients and failed in 3 (6%) patients (patients 7, 47 and 50). In 2 cases (patients 7 and 47) the catheter could not be advanced from the RV to PA and in the other case (patient 50) the catheter could not be advanced from the right atrium (RA) to a stable RV position. All three procedural failures used the femoral venous approach. These patients subsequently underwent conventional X-ray fluoroscopic guided RHC on the same day, with patients 7 and 47 requiring a loop to be formed in the RA and patients 47 and 50 also necessitating the use of a Nitinol guidewire. CMR-conditional guidewires (Nano4Imaging = 1, EPFlex = 5) were used in six patients without complication. In two of these patients, the procedure ultimately failed and in the other four it succeeded. Real-time cine imaging was required in a total of nine (19%) patients due to difficulties in breath-holding cine acquisition, only one of whom had received intravenous sedation. In addition, four patients (8%; cases 6, 15, 17 and 23) had additional clinically indicated tissue characterisation assessments performed, one of whom (subject #6) included late gadolinium enhancement imaging and extracellular volume quantification at 15-min post-contrast administration. The only two catheter-related adverse events comprised asymptomatic hypotension in two patients (cases 3 and 47) that corrected immediately with intravenous crystalloid whilst in the scanner bore.

Metrics that were significantly associated with guidewire use or procedural failure were those that reflected the severity of PAH, right heart dilatation, RV hypertrophy and RV dysfunction (Table 3). All three patients in whom CMR-RHC was unsuccessful were in WHO functional class IV and had severe pre-capillary PAH with significantly dilated and impaired right hearts (Table 4). The composite endpoint of procedural failure or guidewire use was not associated with procedure number, cardiac output or tricuspid or pulmonary regurgitant fraction.

**Table 3:** Time data

Variable (minutes)	Finding	Range (min-max)
Catheter time (min)	3.6 (2.0–7.7)	0.6–15.1
Catheter and flows time (min)	15.0 (10.9–17.8)	8.0–28.1
Study time (min)	31.7 +/− 7.8	19.0–49.8
Department time (min)	60.0 (54.0–68.5)	45.0–92.0

All normally distributed data are presented as mean ± standard deviation, non-parametric data are presented as median (Q1-Q3)

**Table 4:** Association with guidewire use and procedural failure

Variable	OR	*P*
RVESV	1.028 (1.005–1.053)	0.018*
RVEDV	1.019 (0.997–1.042)	0.089
RVSV	0.973 (0.928–1.019)	0.24
RVEF	0.914 (0.852–0.982)	0.014*
RV mass	1.050 (1.014–1.088)	0.0067^†^
RA area	1.178 (1.020–1.361)	0.026*
Tricuspid RF	1.056 (0.984–1.133)	0.132
Pulmonary RF	1.146 (0.967–1.357)	0.116
RVSP	1.075 (1.014–1.139)	0.015*
mPAP	1.125 (1.020–1.240)	0.018*
TPG	1.111 (1.021–1.209)	0.014*
CO	0.614 (0.303–1.245)	0.18
PVR	1.005 (1.001–1.009)	0.0061^†^

*CO* Cardiac output, *EDV* End diastolic volume, *EF* Ejection fraction, *ESV* End systolic volume, *mPAP* Mean pulmonary artery pressure, *OR* Odds ratio, *PVR* Pulmonary vascular resistance, *RA* Right atrium, *RF* Regurgitant fraction, *RV* Right ventricle,* RVEDV* right ventricular end-diastolic volume, *RVEF* right ventricular ejection fraction, *RVESV* right ventricular end-systolic volume, *RVSP* Right ventricular systolic pressure, *SV* Stroke volume, *TPG* Transpulmonary gradient

*P < 0.05, †*P* < 0.01

### Procedural duration

Procedural timings are summarized in Table 5, with a median catheter time of 3.6 (2.0–7.7) minutes (range 0.6–15.1). The mean time to complete the full CMR-RHC protocol (including catheter advancement, great vessel flow assessment and biventricular cine stack) was 31.7 ± 7.8 min (range 19.0–49.8). The median total department time (CMR-RHC plus patient preparation and transfer time) was 60.0 (range 54.0–68.5) minutes (range 45.0–92.0), respectively. There were no significant differences in any of the four timed components of the study with or without sedation use.

**Table 5:** Characteristics of patients who had procedural failure

Variable	Case 7	Case 47	Case 50
WHO FC	4	4	4
RVEDV (mL)	200	193	146
RVESV (mL)	159	141	87
RVEF (%)	20	27	40
RV mass (g)	142	123	87
RA area (cm^2^)	33	25	31
RVSP (mmHg)	65	82	92
mPAP (mmHg)	58	50	52

*EDV* End diastolic volume, *EF* Ejection fraction, *ESV* End systolic volume, *mPAP* Mean pulmonary artery pressure, *RA* Right atrium, *RV* Right ventricle, *RVSP* Right ventricular systolic pressure, *WHO FC* World Health Organisation Functional Class

**Fig. 5:**
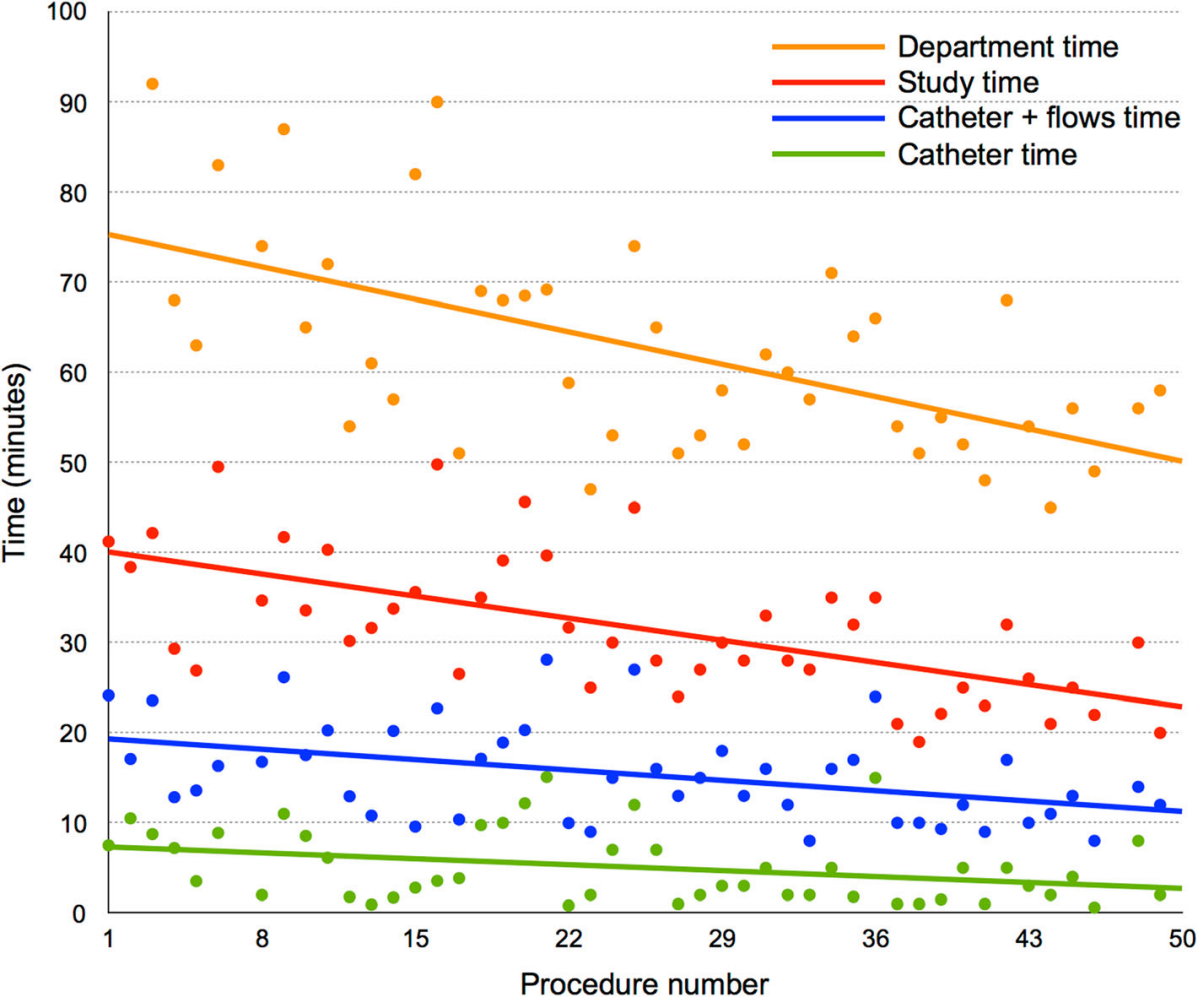
Procedure times plotted against procedure number. All times became significantly shorter with increasing procedural experience (*p* < 0.05). Department times were unavailable for the first two cases as this component of the procedural workflow was not originally part of the study protocol. As such, department times were only recorded after the first two cases had taken place

Times plotted against procedure number are shown in Fig. 5, with all times becoming significantly shorter with increasing procedural experience (*p* < 0.05). However, it can be seen that total department time fell more quickly than catheter time or total procedure time. Severe PH, right heart dilatation, RV hypertrophy and RV dysfunction were all associated with longer catheter times (Table 6). Catheter time was not associated with cardiac output or tricuspid or pulmonary regurgitant fraction.

**Table 6:** Association with catheter time

Variable	rho	*P*
RVESV	0.41	0.0043^†^
RVEDV	0.41	0.0047^†^
RVSV	0.12	0.44
RVEF	−0.32	0.031*
RV mass	0.43	0.0025^†^
RA area	0.38	0.0090^†^
Tricuspid RF	−0.023	0.88
Pulmonary RF	−0.24	0.11
RVSP	0.46	0.0013^†^
mPAP	0.42	0.0030^†^
TPG	0.36	0.013*
CO	0.21	0.15
PVR	0.24	0.10

*CO* Cardiac output, *EDV* End diastolic volume, *EF* Ejection fraction, *ESV* End systolic volume, *mPAP* Mean pulmonary artery pressure, *PVR* Pulmonary vascular resistance, *RA* Right atrium, *RF* Regurgitant fraction, *RV* Right ventricle, *RVEDV* right ventricular end-diastolic volume, *RVEF* right ventricular ejection fraction, *RVESV* right ventricular end-systolic volume, *RVSP* Right ventricular systolic pressure, *SV* Stroke volume, *TPG* Transpulmonary gradient

*P < 0.05, †*P* < 0.01

## Discussion

The main findings of this study were: 1) CMR-RHC is feasible within a normal clinical workflow with standard technology in an adult population of patients with suspected or known PAH, 2) Procedural failure is rare, but more likely in patients with severe disease (both in terms of PA pressure and right heart chamber dilatation), 3) Procedure time increases with worsening disease but is still in the clinically acceptable range. Overall, this study demonstrates that CMR-RHC can be safely performed in patients with adverse cardiopulmonary hemodynamics and incorporated into routine clinical practice without the need for expensive extra infrastructure. We have shown that CMR-RHC has comparable procedure times to a standard CMR examination but with the additional hemodynamic information provided by invasive right heart catheterization, all in a single procedure.

CMR-guided RHC is not a new concept [4–6] with recent work demonstrating the feasibility of the technique in large groups of patients [7]. Furthermore, measurement of cardiac output using phase contrast CMR has previously been validated against the Fick principle, facilitating a robust method to assess PVR by CMR-RHC [6, 7]. However, previous studies have not focused on CMR-RHC in acquired PAH, particularly patients with more severe disease. This is the first study of CMR-RHC in a large group of patients attending for the evaluation of suspected or known PAH. The majority of our patients were in WHO functional class 3 and are representative of the overall clinical population referred for RHC at a large adult PAH center. The technique was feasible in the majority of patients, suggesting that it has a role as a routine clinical investigation in this setting.

### Procedural failure and guidewire usage

Only three out of 50 RHC cases were unsuccessful in the CMR environment, although in six instances a guidewire was used with two of these still ending in failure. Due to the significant overlap between guidewire usage and failure, we used a composite outcome of guidewire use or failure to investigate hemodynamic and CMR associations. The main measures associated with guidewire use or failure were higher PA pressures and increasing RV dilation, hypertrophy and dysfunction. In fact, all three patients in whom the procedure failed had significantly deranged pulmonary hemodynamics and severely hypertrophied, dilated and impaired right hearts. The relationship between right heart size and failure or guidewire use is unsurprising as catheter manipulation does become more difficult in dilated chambers, particularly if systolic function is reduced. We also found that the catheter can become lodged in the thickened trabeculations of the hypertrophied RV, explaining the association with RV mass. It is likely that higher PA pressures associated with guidewire use or failure are a surrogate for right heart chamber dilatation, rather than representing a direct effect on catheter manipulation.

In the conventional cardiac catheterization laboratory, these obstacles are overcome by using a metallic guidewire, creating catheter loops in the RA and/or by using internal jugular venous access. We did have access to CMR-conditional guidewires, with a successful outcome in four cases. However, these devices lack the mechanical properties of their metallic equivalents and currently only have discrete markers towards the guidewire tip for visualization. This is in comparison to metallic guidewires that can be visualized along their whole length using X-ray fluoroscopy. Furthermore, only the catheter balloon is visible in CMR whilst the catheter shaft is not seen. We believe that these combined limitations in guidewire and catheter shaft visualization preclude the safe formation of catheter loops using CMR in a conventional CMR scanner environment in which immediate X-ray fluoroscopic back-up is unavailable. Improvements in guidewire technology may overcome these limitations, and recent work has also focused on improving catheter shaft visibility using nitinol-based guidewires in the CMR environment under low-specific absorption rate (SAR) CMR protocols [10]. Overall, our early experience suggests that procedural failure using femoral venous access is uncommon even using standard equipment and was confined to WHO functional class 4 patients with PH. Alternative approaches could be considered for this group of patients attending for CMR-RHC, including the use of different CMR-safe catheters (pre-shaped balloon-tipped or non-balloon-tipped, with or without CMR-conditional guidewires) and internal jugular venous access.

### Procedure time

One of the main arguments against CMR-RHC has been long procedure times. However, we demonstrate that catheterization and total procedure times were relatively short. Each patient only occupied the department for approximately 1 hour, allowing 3–4 cases to be performed in a single half-day session. Furthermore, CMR-RHC not only provides measures of PA pressure and PVR, but also of biventricular size and function. Therefore, we believe that CMR-RHC is a feasible and time efficient method of physiologically assessing patients with PH. The catheter time modestly improved with procedure number, reflecting a learning curve with advancing a catheter under CMR-guidance. However, total procedure and department times fell by an even greater amount with increasing procedural experience. This suggests that the main improvements come from improved workflow rather than faster catheterization. Study times could be further improved with the use of accelerated imaging for the acquisition of great vessel flows and cine images. However, the aim of this study was to demonstrate the feasibility of CMR-RHC using standard commercially available hardware and software in order to disseminate the technique without the need for non-standard equipment and sequences.

To our knowledge, this is the first study to investigate the association between catheterization time and hemodynamic parameters. As with procedure failure, the main associations were higher PA pressures and increasing RV dilation, hypertrophy and dysfunction. The reasons for these associations are similar to previously described for failure and guidewire usage. This suggests that it might be useful to ensure a mix of disease severity when performing multiple cases in the same session.

Combining hemodynamic and CMR data provides a comprehensive assessment of patients under investigation for PAH in a single study. However, these data can routinely be obtained at separate appointments. Our CMR-RHC procedure times are faster than the total duration of the component investigations performed individually. This confers potential advantages that include cost savings and faster clinical workflows, benefitting both patients and the institution. The times reported in this study included the learning curve that is inherent in setting up a clinical CMR-RHC service. Analyses of the impact of an established CMR-RHC program on cost and patient experience would be worthwhile in evaluating the clinical utility of this combined approach in a tertiary PAH referral center.

### Limitations

Patients routinely had CMR-RHC performed via the femoral approach as per our standard institution protocol. However, the buoyancy and natural upward-pointing arc of the air-filled balloon catheter via the internal jugular venous approach could be advantageous in more technically challenging cases, and merits further study. Furthermore, the use of alternative commercially available CMR-safe curved-tip catheters may have offered greater potential for procedural success in these cases [7]. Whilst the air-filled balloon was adequately visualized in our cohort of patients, previous studies have shown that gadolinium-filled balloons were more consistently conspicuous especially when using a saturation-preparation sequence [4]. Air-filled balloons have the advantages of greater buoyancy and more favourable inflation characteristics, but the enhanced visibility of gadolinium-filled balloons may be advantageous in cases of more complex anatomy that were not encountered in our cohort. Similarly, dedicated interventional CMR workstations that offer simultaneous real-time visualization of multiple scan planes may be beneficial in such complex cases. Our set-up utilized the scanner headphones to communicate between catheter and scanner operators, with the patient receiving regular verbal checks by nursing staff and also holding the emergency alarm to signal if they needed to communicate. This approach worked well for our short CMR-RHC studies, but commercially available systems that enable communication amongst circulating staff and with patients in the CMR environment provide an alternative solution [4, 7]. These systems may be of particular use in complex interventional CMR, but at an additional cost to purchase. We did not record equivalent time parameters for patients undergoing RHC under X-ray-fluoroscopic guidance as a comparator group. However, our CMR-RHC procedure times are nevertheless of a similar order of magnitude to a conventional cardiac catheterization laboratory. Our patient cohort was also not large enough to perform multivariable analysis of the predictors of guidewire usage and procedural failure. However, the factors identified in this study are likely related to one another, and, as such, provide a signal as to the scenarios in which CMR-RHC may be more challenging.

## Conclusions

CMR-guided RHC is a safe and feasible technique for the investigation of PAH that can be performed within a normal clinical CMR environment. Our approach using standard CMR technology is widely applicable and generalizable, allowing dissemination of the technique to other PAH centres. The favorable CMR-RHC procedure times allow a more comprehensive patient assessment to be performed within the timeframe of a routine clinical CMR workflow, conferring potential financial savings and improvements in clinical productivity. Using CMR for the simultaneous measurement of pulmonary pressures and cardiac volumes also has great potential for advancing our understanding of PAH, providing a platform to investigate potential novel biomarkers that reflect the simultaneous assessment of the right heart and pulmonary circulation.

## Additional files

Additional file 1: **Video S1** Patient preparation. The patient lays on the scanner table to have surface vector-cardiogram electrodes attached and a phased array body coil positioned. The scanner table is then undocked and transferred to the preparation area outside the scanner room. **Video S2** Vascular access. The venous sheath is inserted under local anaesthetic using either manual palpation or under ultrasound guidance. The patient is connected to an electrocardiogram (ECG) monitor and a blood pressure monitor during the sheath insertion to monitor for vasovagal events. **Video S3** Drape wrap. The sterile drape is folded over onto the patient firstly from the operator side and subsequently from the opposite side of the table (the latter fold using the underside of the drape) in order to maintain sterility of the operating field. **Video S4** Transfer to scanner. The patient is transferred back into the CMR scanner room. At the same time as vascular access is obtained, the side of the scanner and the inner curvature of the bore on the side of the operator (the side of venous access) are draped. A CMR-compatible non-invasive blood pressure cuff and a peripheral oxygen saturation finger probe are attached to the patient prior to being positioned inside the scanner bore. (MOV 18383 kb)

## Data Availability

The datasets used and/or analysed during the current study are available from the corresponding author on reasonable request.

## Abbreviations

BSA: Body surface area
bSSFP: Balanced steady state free precession
CMR: Cardiovascular magnetic resonance
CO: Cardiac output
CTD: Connective tissue disease
EDV: End-diastolic volume
EF: Ejection fraction
ESV: End systolic volume
LA: Long axis
LV: Left ventricle/left ventricular
mPAP: Mean pulmonary artery pressure
PA: Pulmonary artery
PAH: Pulmonary artery hypertension
PCWP: Pulmonary capillary wedge pressure
PVOD: Pulmonary veno-occlusive diseases
PVR: Pulmonary vascular resistance
RA: Right atrium/right atrial
RHC: Right heart catheterization
RV: Right ventricle/right ventricular
RVSP: Right ventricular systolic pressure
TPG: Transpulmonary gradient
VCG: Vector cardiogram
WHO: World Health Organisation
WHO-FC: World Health Organization functional class
